# Dataset on discovery of microRNAs in *Centella asiatica* (L.) Urb.

**DOI:** 10.1016/j.dib.2020.106451

**Published:** 2020-10-22

**Authors:** Gouri Priya Ranjith, Jisha Satheeshan, K.K. Sabu

**Affiliations:** Jawaharlal Nehru Tropical Botanic Garden and Research Institute^⁎⁎^, Palode, Thiruvananthapuram 695562, India

**Keywords:** miRNA, *Centella asiatica*, Centelloside, Illumina, Medicinal plant

## Abstract

MicroRNAs (miRNAs) are small (21–24 nucleotides), non-coding, riboregulators that regulate gene expression in eukaryotes. Pentacyclic triterpenoid saponins and sapogenins known as centellosides of the plant *Centella asiatica* (L.) Urb. are known for their broad spectrum medicinal properties. Two *C. asiatica* accessions viz.,CA301 and CA308 were selected for the miRNAome profiling. Total RNA isolated from fresh young leaves of both accessions along with their replicas was used for library construction. Illumina® sequencing of the four small RNA libraries generated a total of 59,234,923; 58,487,817; 59,520,376; 64,093,228 raw reads. The raw reads were quality filtered and used for the prediction of conserved and novel miRNAs. A total of 227 conserved and 109 novel microRNAs were identified from the libraries. Target gene prediction done using psRNAtarget and PANTHER™GO helped in localization of predicted targets. KEGG (Kyoto Encyclopedia for Genes and Genomes) was used for pathway prediction of the targets of predicted miRNAs. The present study provides first elaborated glimpse of miRNA pool of *C. asiatica.* The outcome of this research could help understand miRNA dependent regulation of centelloside biosynthesis and to design further metabolic engineering experiments to enhance their content in this important medicinal plant.

## Specifications Table

**Table:** 

Subject	Agricultural and Biological Sciences
Specific subject area	Plant Science
Type of data	Text (FASTQ sequence files), table
How data were acquired	High-throughput RNA sequencing using Illumina HiSeq 2500 Sequencing System
Data format	Raw data in FASTQ file
Parameters for data collection	Total RNA isolation from leaves of *Centella asiatica* (L.) Urb. Small RNA sequencing using Illumina HiSeq 2500 Sequencing platform
Description of data collection	Freshly collected tender leaves were used for RNA isolation, Small RNA seq libraries representing two accessions of *Centella* along with their replicas were sequenced for discovering known and novel miRNAs
Data source location	1) CA301 Leaf Palode, Thiruvnanthapuram, Kerala N8°45’10” E77°01’41” 2) CA308 Leaf Bonaccord, Thiruvnanthapuram, Kerala N08°41’15” E77°10’52” 30-12-2015
Data accessibility	Repository name: NCBI SRA Data identification number: PRJNA553029 Direct URL to data: https://www.ncbi.nlm.nih.gov/sra/PRJNA553029

## Value of the Data

• The data represents profiling of microRNAs of the plant *Centella asiatica* where a correlation between miRNA and secondary metabolism of the high valued medicinal plant was analyzed.
• The identified miRNAs were used to predict their potential roles in regulating genes involved in centelloside biosynthetic pathway specifically.
• The findings could advance our understanding of the regulatory mechanism of miRNAs in centelloside biosynthesis which could facilitate a miRNA-based biotechnology for manipulating centelloside production in both *in vitro* and *in vivo* systems.
• Expression profiles are available in the form of raw sequencing reads that can be further processed by researchers using their own bioinformatic pipeline.

## 1 Data Description

The dataset contains raw sequencing data obtained through the small RNA sequencing of leaf tissue of two accessions CA301 and CA308 of the medicinal plant *Centella asiatica* with their duplicates. The data files (reads in FASTQ format) were deposited at NCBI SRA database under project accession PRJNA553029. The summary of the processed data and assembly statistics are shown in Table 1 and the details of gene ontology classification of the predicted targets of all miRNAs discovered from the dataset are presented in Table 2.

**Table 1:** Read and assembly statistics

Plant accession	CA301_R1	CA301_R2	CA308_R1	CA308_R2
Raw reads	59,234,923	58,487,817	59,520,376	64,093,228
Reads after 3’ and 5’ adaptors	12,092,259	12,133,090	11,547,521	10,991,576
Reads after removal of other non-coding RNAs	7,676,703	8,565,464	7,178,504	5,820,407
Unique reads after transcriptome alignment	7812970	8433792	7472283	6576025
Reads matching against known miRNAs aligned to miRBase database	2101	2264	1964	1505
Number of known miRNAs	185	189	179	168
Reads retained for novel miRNA prediction	7077406	7670195	6762007	6084399
Novel miRNAs predicted	54	56	38	21

**Table 2:** Gene Ontology classification of predicted targets of total conserved miRNAs

GO Terms	Number Of Functional Hits
Biological process	3558
Cellular Component	2910
Molecular Function	3208
Protein Class	2934

## 2 Experimental Design, Materials, and Methods

### 2.1 Plant material

Total RNA was isolated from the fresh young leaves of two *C. asiatica* accessions viz., CA301 and CA308, collected from natural populations located at JNTBGRI and Bonaccord, Thiruvananthapuram, India. The tissues were immediately frozen and stored in liquid nitrogen, until processing.

### 2.2 Total RNA isolation and small RNA sequencing

Total RNA was isolated from the young leaves of both accessions along with their replicas using a modified miRNeasy Mini Kit + Trizol method. Quantity and quality analysis of the samples were done using 4200 TapeStation System (Agilent Technologies). Four sets of total RNA enriched with miRNAs were purified and cDNA library was constructed using Illumina® TruSeq® Small RNA Library Prep Kit according to manufacturer's instructions. The purified libraries were subjected to small RNA sequencing using Illumina® HiSeq 2500 sequencer.

### 2.3 Identification of conserved and novel miRNAs

miRNA identification and target prediction were done according to earlier report [1]. The raw reads were preprocessed to remove adapter sequences, rRNAs, tRNAs, other small RNAs and sequences below 17 and 25 nt. The identical sequences were collapsed using FASTQ/A Collapser tool available in the FASTX-Toolkit and the known miRNAs were identified by performing blastn search against plant mature miRNAs from miRBase database. The remaining reads were mapped onto the transcriptome sequence of *C. asiatica* for the identification of novel miRNAs as the whole genome of *C. asiatica* was not available. The aligned reads were used as input for predicting the novel miRNAs using miRDeep-P software [2]. Gene ontology enrichment was done using PANTHER gene ontology classification tool [3].

### 2.4 Target prediction and pathway annotation

*In silico* target gene prediction of known and novel miRNAs was done using plant miRNA target gene prediction software psRNATarget [4] with default parameters. *Arabidopsis thaliana* (transcripts, removed miRNA gene, TAIR (The Arabidopsis Information Resource) version 10, released on 2010_12_14) was selected from the input list of cDNA library in the psRNATarget tool as the reference source. KEGG databases were utilized to find targets that were putatively involved in terpenoid backbone, mono-, sesqui- and tri- terpene biosynthesis pathways [5].

## CRediT Statement

Kallevettankuzhy Krishnannair Sabu: Conceptualization, Supervision, Reviewing and Editing.

Gouri Priya Ranjith: Methodology, Data curation and Writing- Original draft preparation.

Jisha Satheesan: Visualization, Investigation.

## Declaration of Competing Interest

The authors declare that they have no known competing financial interests or personal relationships that could have appeared to influence the work reported in this paper.
